# Structural Context of Disease-Associated Mutations and Putative Mechanism of Autoinhibition Revealed by X-Ray Crystallographic Analysis of the EZH2-SET Domain

**DOI:** 10.1371/journal.pone.0084147

**Published:** 2013-12-19

**Authors:** Stephen Antonysamy, Bradley Condon, Zhanna Druzina, Jeffrey B. Bonanno, Tarun Gheyi, Feiyu Zhang, Iain MacEwan, Aiping Zhang, Sheela Ashok, Logan Rodgers, Marijane Russell, John Gately Luz

**Affiliations:** 1 Lilly Biotechnology Center San Diego, San Diego, California, United States of America; 2 Department of Biochemistry, Albert Einstein College of Medicine University, Jack and Pearl Resnik Campus, Bronx, New York, United States of America; 3 Department of Radiology, Loma Linda University Medical Center, Loma Linda, California, United States of America; University of Dundee, United Kingdom

## Abstract

The enhancer-of-zeste homolog 2 (EZH2) gene product is an 87 kDa polycomb group (PcG) protein containing a C-terminal methyltransferase SET domain. EZH2, along with binding partners, i.e., EED and SUZ12, upon which it is dependent for activity forms the core of the polycomb repressive complex 2 (PRC2). PRC2 regulates gene silencing by catalyzing the methylation of histone H3 at lysine 27. Both overexpression and mutation of EZH2 are associated with the incidence and aggressiveness of various cancers. The novel crystal structure of the SET domain was determined in order to understand disease-associated EZH2 mutations and derive an explanation for its inactivity independent of complex formation. The 2.00 Å crystal structure reveals that, in its uncomplexed form, the EZH2 C-terminus folds back into the active site blocking engagement with substrate. Furthermore, the *S*-adenosyl-L-methionine (SAM) binding pocket observed in the crystal structure of homologous SET domains is notably absent. This suggests that a conformational change in the EZH2 SET domain, dependent upon complex formation, must take place for cofactor and substrate binding activities to be recapitulated. In addition, the data provide a structural context for clinically significant mutations found in the EZH2 SET domain.

## Introduction

Regulation of gene expression via gene silencing is a critical and conserved mechanism for the management of cellular growth, differentiation, survival, and senescence. Diverse transcriptional processes are regulated by this mechanism, i.e., gene silencing of the mating type loci in *Saccharomyces cerevisiae* [1], the *Drosophila* homeotic gene cluster [2,3], imprinted genes [4] and the inactive X chromosome in mammals [5]. In *Drosophila*, the *Polycomb*-group complexes, PRC1 and PRC2, and the trithorax group protein assemblies act via opposing mechanisms to regulate homeobox (HOX) genes, the former being repressive and the latter being activating, generally. The PRC complexes repress gene expression through the SAM-catalyzed methylation of histone 3 (H3) proteins at lysine 27 (H3K27) [6] as well as other mechanisms [7,8]. Methylation of histone (H3K27) by PRC complexes is believed to be catalyzed by its conserved SET-domain containing member, Enhancer of zeste [E(z)] [9]. EZH2 is the human homolog of E(z), the C-terminal SET domain of which is the most conserved region of the protein by primary sequence [10]. EZH2 isoform A is an 86 kDa, 751 amino acid, multi-domain protein. There are at least five human isoforms. In addition to methylation of H3K27, EZH2 has been shown to methylate cellular proteins [11] and act as a co-activator of steroid hormone receptors [12]. Unlike other SET domains, i.e., SET7, SET8, and SUV39H1, EZH2 is inactive on its own and requires binding partners (SUZ12 [13,14] and EED [15,16]) for activity. 

**Figure 1:**
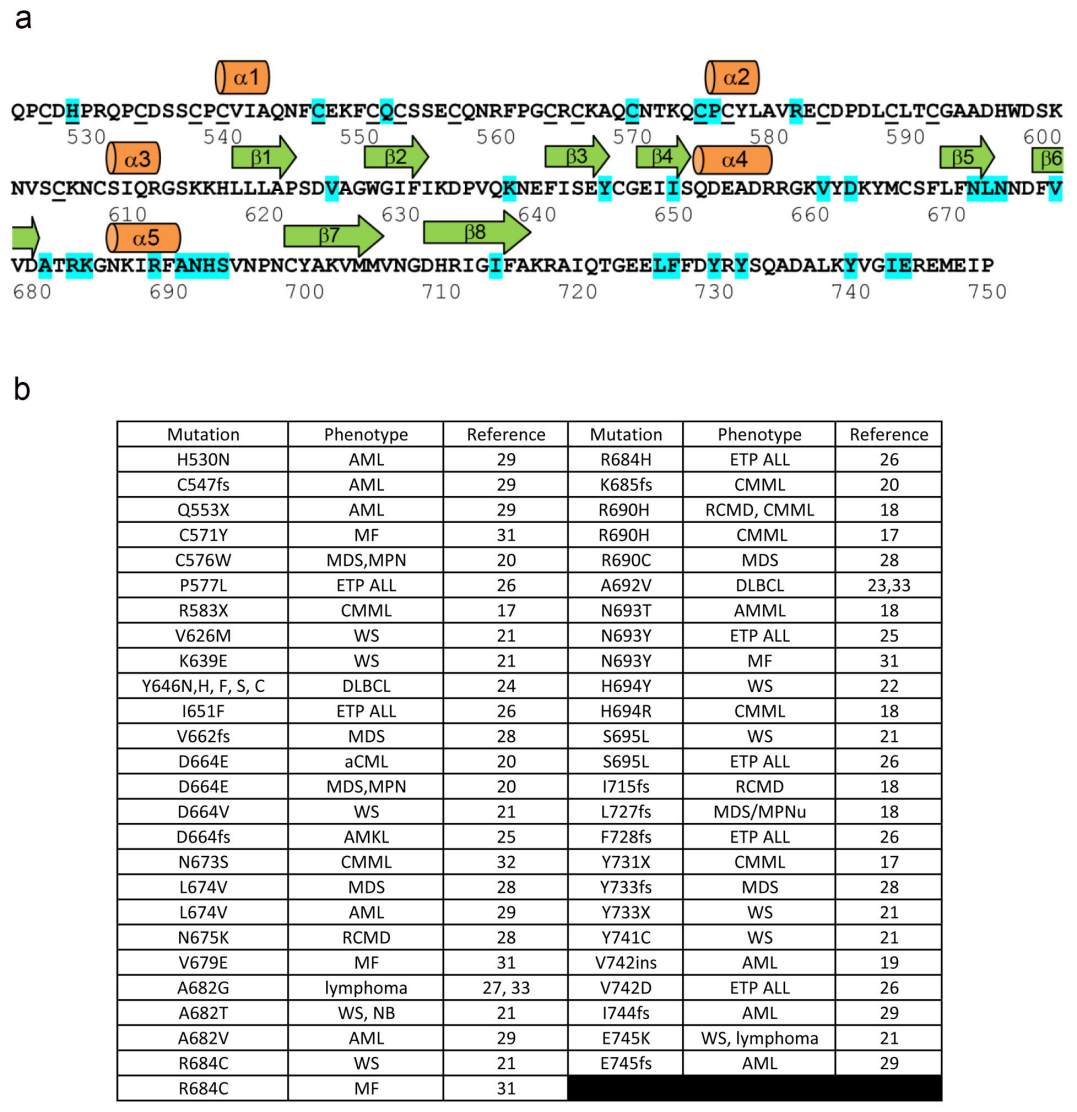
Mutations of the EZH2-SET domain. (a) The amino acid sequence of the EZH2-SET domain is shown with the secondary structure assignments depicted above. Residues which coordinate zinc are underlined. Mutated amino acids identified in association with disease are highlighted cyan. The specific mutations are annotated below with the disease-associated with each mutation, the nature of the mutation, and the reference in which the mutation is described. The sequence is numbered in accordance with EZH2 isoform A and the numbering for some mutations has been transposed from the original references so that all mutations can be referred to relative to the same sequence. (b) Details of mutations delineated in Figure 1a. (Abbreviations: AMKL, acute megakaryoblastic leukemia; AML, acute myeloid leukemia; AMML, acute myelomonocytic leukemia; CMML, chronic myelomonocytic leukemia; DLBCL, diffuse large B-cell lymphoma; ETP ALL, early T-cell precursor acute lymphoblastic leukemia; MDS, myelodysplastic syndrome; MPN, myeloproliferative neoplasms; MPNu, myeloproliferative neoplasms unclassifiable; NB, neuroblastoma; MF, myelofibrosis; RCMD, refractory cytopenia with multilineage dysplasia; WS, Weaver Syndrome; fs, frameshift; X, nonsense).

EZH2 sequence mutations [17-33], expression levels, and copy number aberrations are correlated with the incidence and aggressiveness of various cancers [34-38] and other diseases (Figure 1a, b) [21,22]. Mutation of EZH2 residue Y646 was found in 7% of follicular lymphoma cases [24] and 22% of diffuse large cell B-cell lymphomas and was coincident with increased H3K27 trimethylation. However, other studies have not found a correlation between EZH2 mutation and loss of *in vitro* H3K27 trimethylation in follicular lymphoma [39]. The oncogenic potential of some EZH2 mutations may in part be attributable to a gain-of-function in which the substrate preference of EZH2 is altered such that dimethylated H3K27 becomes the preferred substrate [40,41]. Analogous gain-of-function mutations were detailed structurally and biochemically in previous studies on the SET7/9 domain [42,43]. Histone lysines may be sequentially methylated and are present in non-, mono-, di-, and tri- methylated states. Lysine methylases have varied specificities for each of these reactions, and, similarly, these different methylated states recruit distinct regulatory binding proteins.

EZH2 mutations have also been associated with incidence and poor prognosis in myelodysplastic syndromes [19,20,29]. In contrast to its role in lymphomas, EZH2 appears to act as a tumor suppressor in myeloid dysplasias where the oncogenic potential of its mutation is attributed to loss-of-function with respect to methylation [29]. In addition, upregulation of EZH2 has been linked to glioblastomas [44,45]. One possible glioblastoma-related tumorigenic mechanism involves activation of STAT3 via direct methylation by phosphorylated EZH2 [46]. The rare genetic disorder Weaver Syndrome (WS) is also associated with mutations in EZH2 [21,22].

Due to the clearly established correlation between EZH2 function and numerous clinical syndromes, significant effort has been expended on the identification and development of specific small molecule inhibitors of the EZH2 methyltransferase activity [47-49]. Two such inhibitors, EPZ005687 [48] and GSK126 [49], have been reported and shown to globally reduce H3K27 trimethylation and inhibit the proliferation of lymphoma cell lines. We determined the crystal structure of the isolated EZH2 SET domain in order to compare and contrast it with the crystal structure of other SET domains [50], better understand the structural context of clinically relevant EZH2 mutations, and to possibly elucidate autoregulatory mechanisms of EZH2 methyltransferase activity. 

## Materials and Methods

### Protein expression, purification, and crystallization

A gene encoding the TEV-cleavable N-terminally his-tagged EZH2 catalytic domain (amino acid residues 526-751, isoform A, accession NP_004447.2) was expressed in *Sf*9 cells using a recombinant baculovirus. Boundaries for the SET domain had been optimized by performing limited proteolysis mass spectrometry (LPMS) on the full-length EZH2 (data not shown). Cell pellets were stored at -80°C and subsequently lysed by incubation with stirring in cold (4°C) lysis buffer containing 0.02 M Tris-HCl pH 8.0, 0.5 M NaCl, 10% Glycerol, 0.025 M imidazole, 5 mM BME, benzonase, and protease inhibitor (Roche complete EDTA-free, cat. 13317600). Cell lysates were clarified by centrifugation (JLA-16.25 @ 16K RPM @ 4 °C), and the his-tagged recombinant protein was purified using Ni-NTA beads (Qiagen) in batch mode. The beads were collected in a drip column after incubation with the decanted supernatant @ 4 °C with stirring and then washed with 20 bed volumes of cold lysis buffer. Recombinant EZH2 was eluted with lysis buffer containing 250 mM imidazole. The eluted sample was dialysed against lysis buffer after the addition of TEV protease. The cleaved sample was reapplied to Ni+ agarose. The flow through fraction containing EZH2 was concentrated and applied to an S200 (GE Healthcare) gel filtration column equilibrated with 10 mM HEPES pH 7.5, 150 mM NaCl, 10% glycerol, and 5 mM DTT. EZH2 containing fractions were pooled and concentrated to 12 mg/ml. The intact mass of the purified protein was confirmed by ESI mass spectrometry. 1 mM ZnCl_2_ and 2 mM S-adenosylhomocysteine were added and the protein was crystallized by sitting drop vapor diffusion using a 1:1 drop ratio (1 µl) against a mother liquor containing 0.1 M MES pH 6.5, 30% PEG MME 5K, 0.2 M (NH_4_)_2_SO_4_. Crystals (space group P2_1_2_1_2_1_; a=45.1 Å, b=57.7 Å; c=75.5 Å;α, β, γ=90°) were cryo-cooled by immersion in liquid N_2_ using 20% ethylene glycol as the cryoprotectant.

### Data collection and structure determination

Datasets were collected at the LRL-CAT beam line at the Advanced Photon Source, Argonne, IL. The structure was determined by SAD phasing, using the anomalous signal from the bound Zn atoms. The Zn atoms were located using Shelx [51], and the structure phased using Mlphare (Collaborative Computational Project 1994). The model building was done with COOT [52], and the structure refined with Refmac [53,54]. Data and refinement statistics are included in Table 1.

**Table 1:** Crystallographic Statistics.

**Data Collection**	
Space Group	P2_1_2_1_2_1_
Cell Dimensions (a,b,c) (Å)	41.1, 57.5, 75.5
Angles (α, β, γ)(o)	90, 90, 90
Resolution (Å)	21.0-2.0 (2.1-2.0) *
Completeness (%)	95.6 (95.6)
Rsym (%)	7.5 (37.2)
Mean I/σ (I)	8.7 (3.3)
Redundancy	3.3 (3.3)
Wavelength (Å)	1.28212
**Refinement**	
Resolution range	2.0-21.0 (2.05-2.00)
Reflections	12465
R_work_	19.8% (21.8%)
R_free_	25.9% (29.2%)
R.m.s. dev. bonds	
lengths (Å)	0.005
angles (**^*o*^**)	0.978
Total # residues	209
Total # protein atoms	1605
Zn	6
SO_4_	1
Total # waters	159
Average B (Å^2^) all	27.8
Peptide	27.0
Zn	25.5
SO_4_	69.7
H_2_O	34.3

(*parenthesis = highest resolution shell)

## Results

### Overall structure

**Figure 2:**
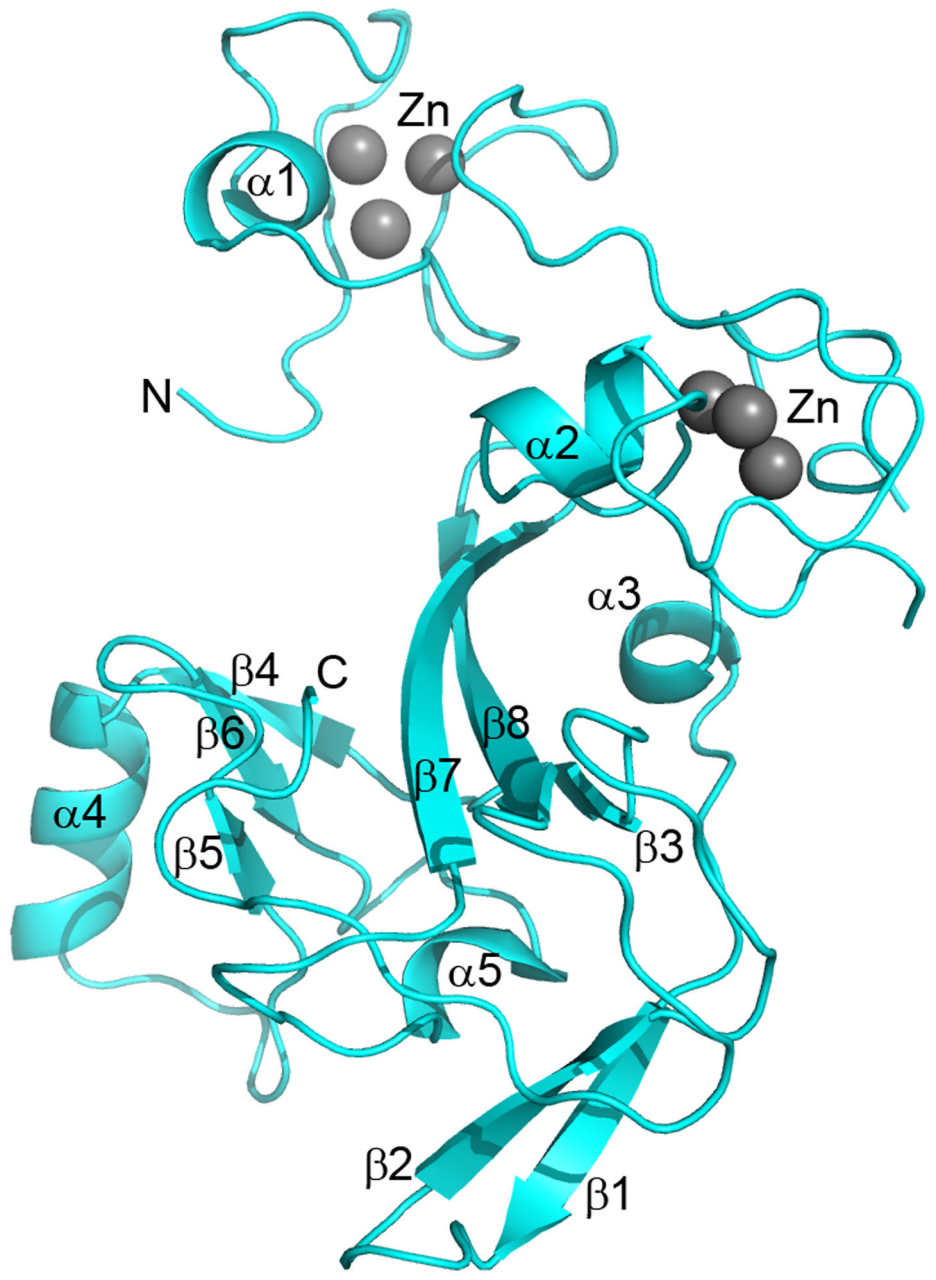
The crystal structure of the EZH2-SET domain. The crystal structure of the EZH2-SET domain is represented as a ribbon model colored cyan. Bound zinc atoms are represented by spheres and colored gray. Secondary structure elements are labeled. The crystal structure contains two N-terminal zinc binding domains each of which binds three zinc molecules. The core of the domain is formed by β-strands 3, 7, and 8. This core is flanked on one side by a three stranded antiparallel β-sheet (β-4, β-6, β-5) and an accessory α-helix (α-4) and bounded below by α-5, β-1, and β-2. The C-terminus turns upward insinuating through the substrate binding cleft between the β-5/β-6 loop and β-7.

EZH2-SET crystallized in the space group P2_1_2_1_2_1_ with one copy per asymmetric unit. The crystal structure (Figure 2) was determined by SAD using the anomalous signal of bound zinc for phasing. After multiple rounds of model building and refinement, the 2.00 Å structure refined to an R_work_ of 19.8% and an R_free_ of 25.9% (Table 1). The final model consisted of one EZH2-SET domain (chain A) containing a total of 209 residues, 6 zinc atoms, 159 water molecules, and 1 sulfate ion. 96.6% of residues are in the most favored region of the Ramachandran plot, and 100% are in the allowed region. The C-terminal residues 737-751 are disordered. Due to lack of representative electron density, internal residues 598-603 and the side chain atoms of residues Q559, D597, V604, K616, D625, K661, D664, K665, and M667 were omitted. For the purposes of this discussion, all residue numbering of EZH2 corresponds to isoform A, and the residue numbers from some references have been transposed such that all residues and mutations can be discussed with reference to their relative position in the same amino acid sequence. 

Found within the pre-SET domain N-terminal to the SET domain, there are two three-atom clusters of bound zinc coordinated by two distinct nine-residue constellations (Figure 2). The first three zinc atoms are coordinated by cysteines 528, 535, 539, 541, 548, 552, 554, and 558 as well as histidine 530. The second set of three zinc atoms is coordinated by cysteines 565, 567, 571, 576, 578, 585, 590, 593, and 606 (Figure 1a). Each zinc binding domain contains a short helical turn which is formed just after the fourth zinc binding cysteine found in the cluster. Both helical turns begin at the proline of C-P-C sequence motifs (Figure 1a). The tertiary structure of the SET domain is similar to that of previously determined SET domain crystal structures. The core of the SET domain is formed by a three-stranded (β-3, β-7, β-8) anti-parallel β-sheet pressed diagonally across a second smaller three-stranded (β-4, β-5, β-6) anti-parallel β-sheet. The opposite face of the smaller β-sheet is decorated by a short α-helix (α-4). A second short α-helix (α-5) is packed against the core on the opposite side by a two-stranded anti-parallel β-sheet (β-1, β-2). Unlike many of its homologs, the C-terminal Post-SET domain of EZH2 does not encode a zinc-binding motif and is largely disordered in the crystal structure. The zinc binding cluster found in the post-SET domain of some homologous methyltransferases helps form the cofactor binding pocket as well as one face of the substrate binding groove [42,43].

### Hypothetical mechanism of auto-inhibition by C-terminus

**Figure 3:**
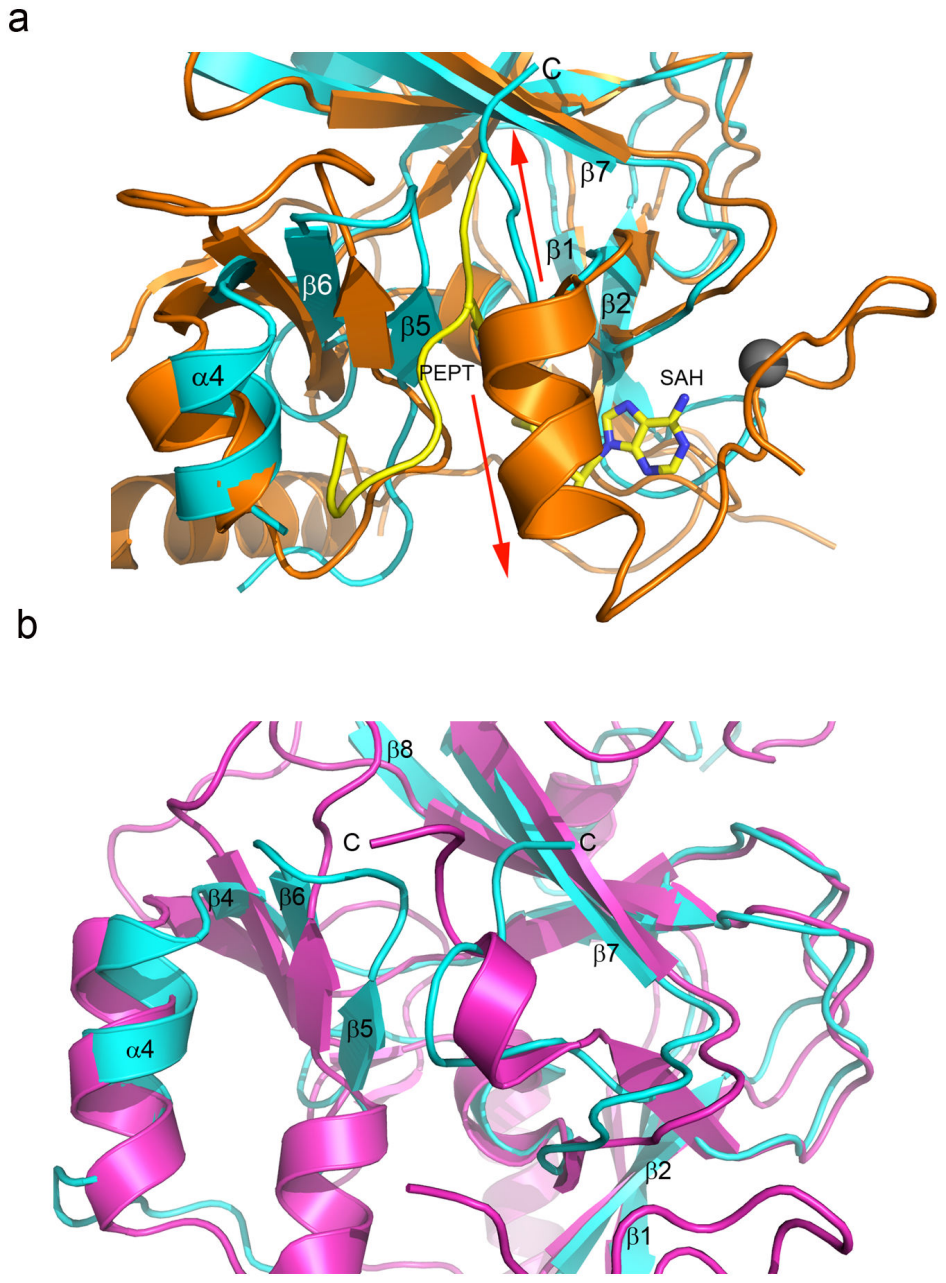
The EZH2-SET domain C-terminus partially occupies the substrate binding groove. (a) The EZH2-SET (cyan) and hEHMT1-SET (orange) (PDB ID:3HNA) domains are superimposed and represented by ribbons. Zinc bound by hEHMT-SET is represented as a gray sphere. The substrate peptide bound by hEHMT1 is a yellow ribbon with the lysine side chain represented as sticks. The SAH bound by hEHMT1-SET is represented by sticks and colored by atom (carbon, yellow; oxygen, red; nitrogen, blue; sulfur, sienna). The C-terminal tail of EZH2-SET turns upwards and occupies the upper region of the substrate binding groove (red arrow pointing up). The C-terminus of hEHMT1-SET turns downward (red arrow pointing downward) forming the lower lobe of the cofactor binding pocket and coordinating one zinc atom. (b) The EZH2-SET (cyan) and SUV39H2 SET domain (magenta) (PDB ID:2R3A) crystal structures are superimposed and represented by ribbons. The C-termini in both structures occupy the collapsed substrate binding groove.

The structural features of EZH2 diverge dramatically from those of most homologous SET domains in the C-terminal, or post-SET, region. (See Figure S1 for sequence alignments). Rather than looping outward and downward after the conserved tyrosine at position 731 to form the lower lobe of the SAM cofactor binding site [50], the C-terminus turns upwards and packs against the loop between β-strands 5 and 6 and the outer edge of β-7 (Figure 3a) with the backbone oxygen of the C-terminal Y731 forming a hydrogen bond with the backbone nitrogen of β-5 N673. Folding back towards the core of the domain, S734 of the C-terminal tail forms three additional hydrogen bonds, one between its backbone carbonyl and the A702 (β-7) amide nitrogen and two between its side-chain hydroxyl and backbone nitrogen and the backbone carbonyl oxygen of N673. Backbone to backbone hydrogen bonds are also formed between the C-terminal tail and the core domain involving the following residue pairs: N735/L674 and A736/A702. The carboxylate of D730 forms a hydrogen bond with the H684 imidazole while additionally stabilizing the conformation of the C-terminal tail coil by forming a salt bridge with the R732 side chain. β5 and the loop following from the left hand boundary of the substrate binding channel and, thusly, the C-terminus sterically blocks substrate binding by occupying the upper groove of the peptide binding site. When compared with structurally homologous SET domains, it appears that the conformation of the C-terminus in this position would impair both cofactor and substrate binding. The conformation is in part reminiscent of that observed in the SUV39H2 SET domain (PDB ID: 2R3A)[50](Figure 3b) in which the empty substrate pocket is collapsed and occupied by a segment of the post-SET domain, while still binding cofactor.

Despite efforts to demonstrate by SPR binding of the isolated EZH2-SET domain to cofactor, cofactor mimics, inhibitors, and substrates, no binding event was ever observed (data not shown). Similarly, although numerous EZH2-SET domain constructs with different boundaries were crystallized in the presence of SAM, SAH, and substrate and yielded structures, electron density maps for the refined models did not provide any evidence of ligand binding.

### Structural Context of Clinically Relevant Mutations in the EZH2-SET domain

#### Mutations of the active site

**Figure 4:**
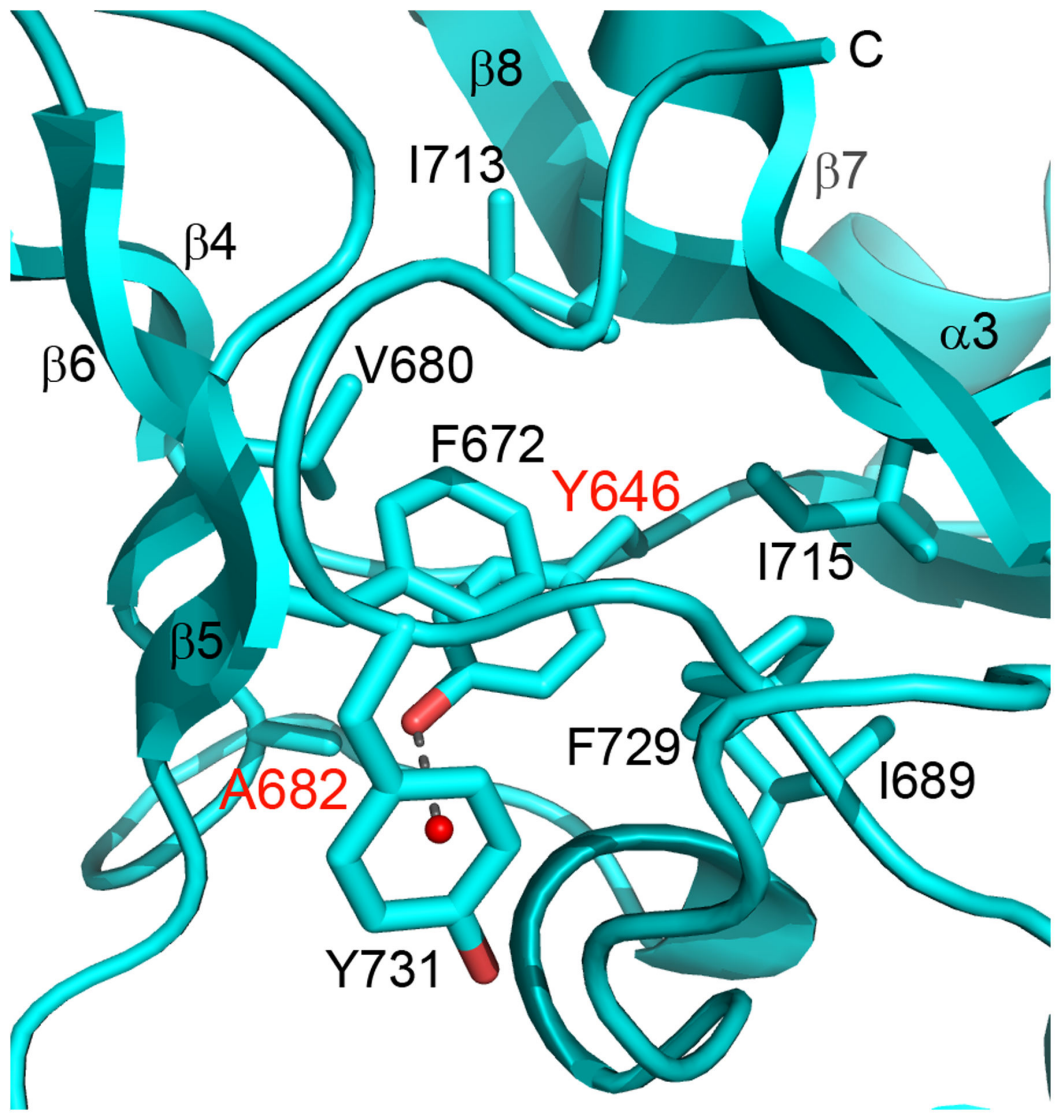
Structural context of Y646 and A682 mutations. The crystal structure of the EZH2-SET domain is represented as a ribbon model (cyan). Side chains are represented as sticks colored by atom (carbon, cyan; oxygen, red). Secondary structure elements are labeled. Y646 is completely buried in a hydrophobic cluster except for the solvent exposed tip of the phenyl ring where the phenyl oxygen forms a hydrogen bond with a water molecule. A682 is packed against the Y646 side chain distal to the catalytic site. Mutation of A682 likely indirectly affects substrate specificity by influencing the conformation of Y646 in the active state. Y646 and A682 mutations have been found in lymphoma [24,27,33], WS [21], and AML [29].

The diffuse large B-cell lymphoma (DLBCL) mutation Y646 [24] is located in the active site of the SET domain with the side chains pointing inwards towards the putative catalytic locus. The Y646 side chain forms only hydrophobic and van der Waals interactions with the surrounding protein atoms while the side chain hydroxyl forms a lone hydrogen bond with a water molecule (9)(Figure 4). The Y646, F672, F729, and Y731 aromatic side chains form a tightly packed hydrophobic core which occludes the lysine substrate binding channel. A shell of hydrophobic residues (V680, A682, I689, I713, and I715) surrounds this aromatic core. It is difficult to determine from the inactive state of the crystal structure how these Y646 mutations (Y>F, Y>H, Y>S, Y>N, Y>C) manifest the trimethylating gain-of-function activity ascribed to them [40]. Curiously, although the substituted amino acids have little in common chemically, they all seem to result in a related phenotype. Perhaps the reduction in space occupied by the substituted amino acid side chains is the common characteristic that allows for increased productive binding of methylated substrates. Mutation of the homologous residue in human SET 7/9 (Y245) to an alanine altered substrate specificity from a mono- to a di-methyltransferase [42]. In the case of EZH2-SET, the burying of the Y646 in the inactive conformation means that numerous internal contacts must be broken in order for the transition to the active state to occur, and mutation of Y646 would therefore necessarily alter the dynamics of this process.

**Figure 5:**
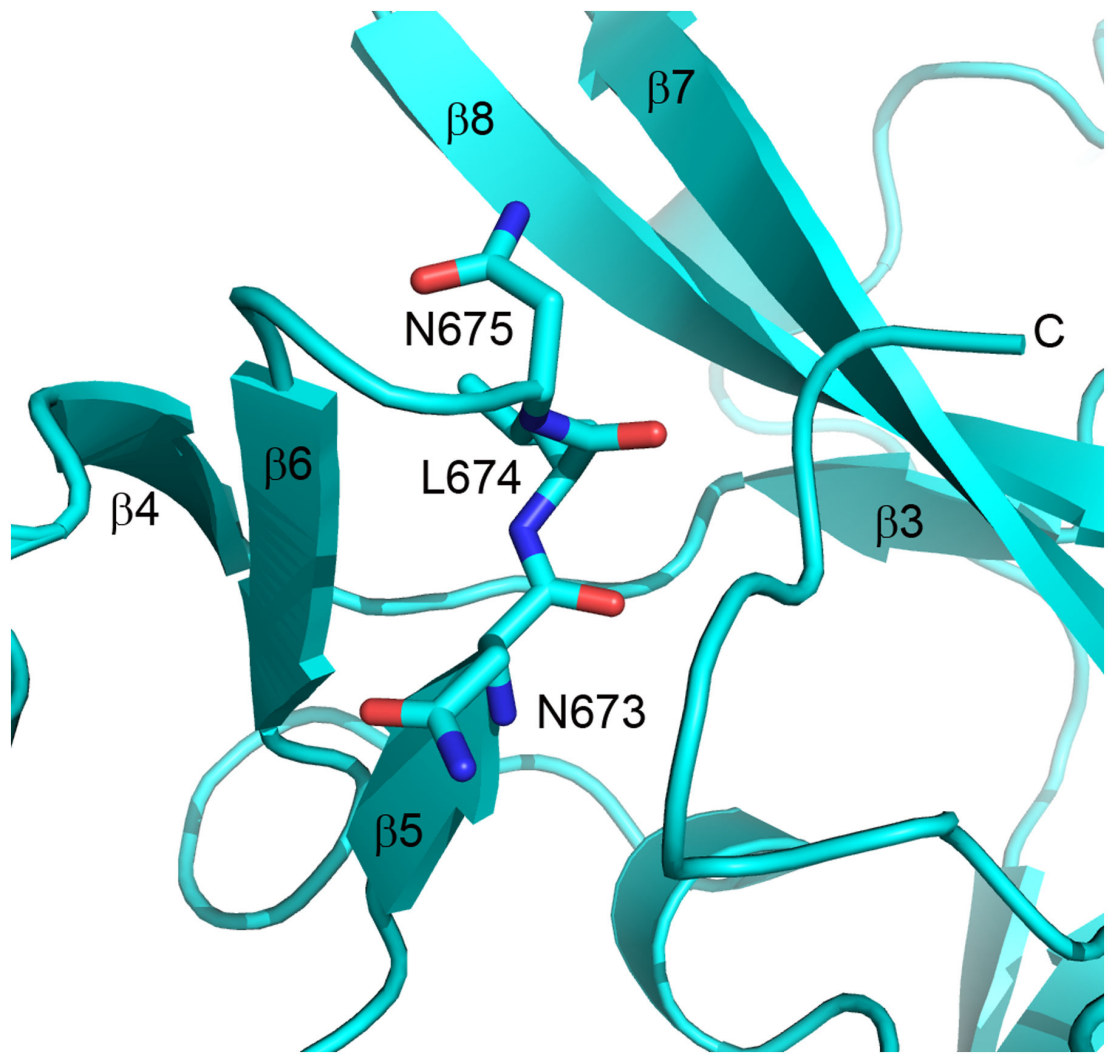
Mutations in the β-5/β-6 loop of EZH2-SET are contiguous with the putative substrate binding cleft. The crystal structure of the EZH2-SET domain is represented as a ribbon model (cyan). Side chains are represented as sticks colored by atom (carbon, cyan; oxygen, red; nitrogen, blue). Secondary structure elements are labeled. N673, L674, and N675 all interact directly with the C-terminal tail which occupies the substrate binding groove. Mutation of these residues could potentially affect substrate binding in the active state as well as the transition from the inactive to active state. An N673S mutation has been identified in CMML [32]. L674V mutations have been found in both MDS [28] and AML [29]. An N675K mutation was discovered in RCMD [28].

An N673S mutation has been found in a case of chronic myelomonocytic leukemia (CMML) [32]. N673 is found at the end of β-5 at the left hand boundary of the substrate binding cleft (Figure 5). The residue found at the analogous position in SET/peptide crystal structures forms critical interactions with the substrate [50]. In the present crystal structure, N673 interacts directly through hydrogen bonding with the trapped C-terminal tail. Mutation of this residue in EZH2-SET may affect both substrate binding and the transition from the inactive to the active state. Similarly, mutations at L674 {(myelodysplastic syndrome, MDS)(L>V)[28]} {(acute myeloid leukemia, AML) (L>V) [29]} and N675 {(refractory cytopenia with multilineage dysplasia, RCMD), (N>K), [28]} appear to have the potential to affect both substrate binding and transformation to the active state (Figure 5). 

**Figure 6:**
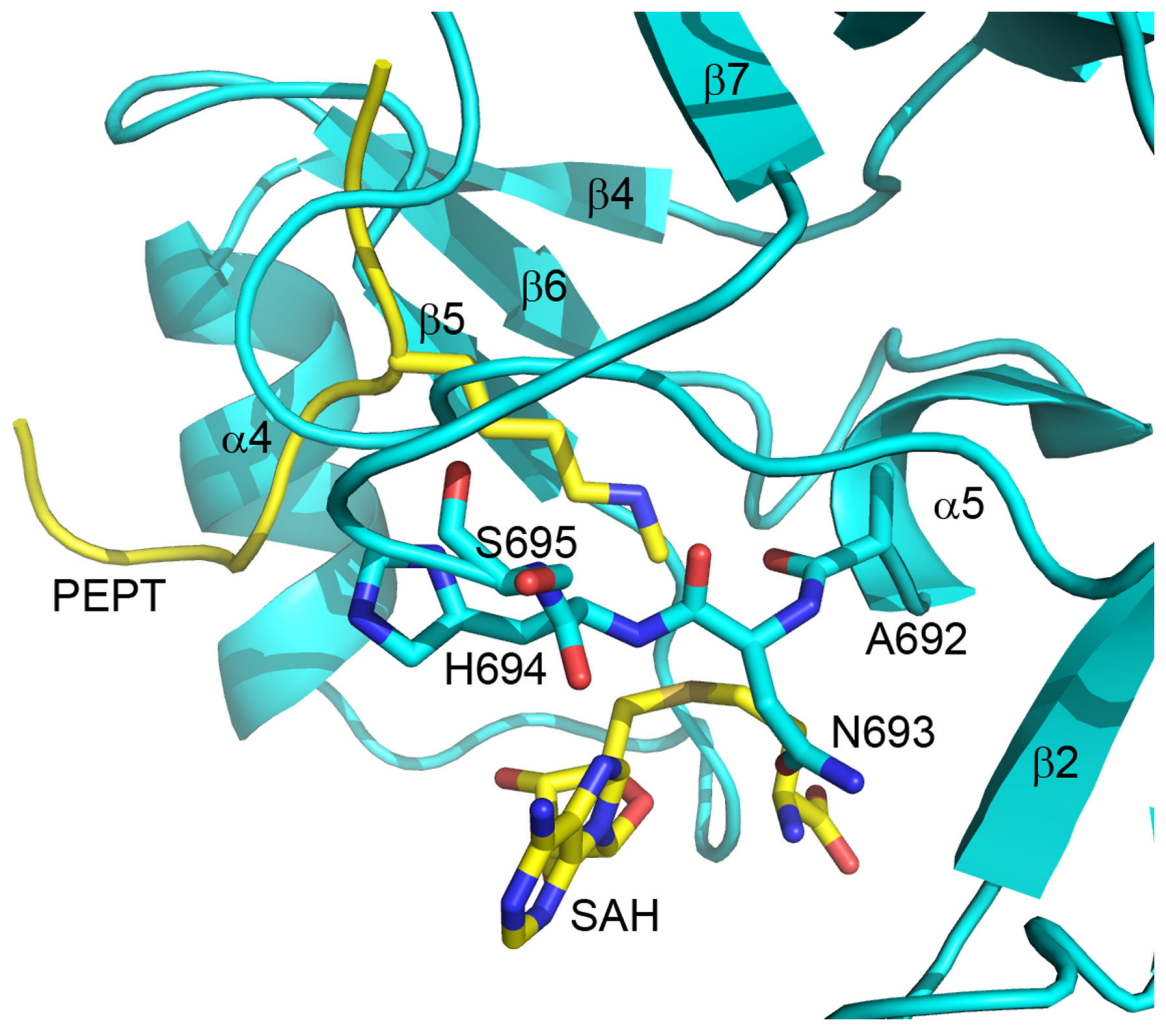
EZH2-SET mutations that may affect cofactor binding. The crystal structure of the EZH2-SET domain is represented as a ribbon model (cyan) with the hypothetical positions of cofactor and substrate (sticks colored by atom: carbon, yellow; oxygen, red; nitrogen, blue) extracted from the superimposed structure of EHMT1/PEPTIDE/SAH (PDB ID: 3HNA). EZH2-SET amino acid side chains are represented as sticks colored by atom (C, cyan; O, red; N, nitrogen). Secondary structure elements are labeled. Mutations at positions A692 {(DLBCL) (A>V) [23,33]}, N693 {(AMML) (N>T) [18]; (ETP ALL) (N>Y) [26]; (MF) (N>Y) [31]}, and H694 {(WS) (H>Y) [22]; (CMML) (H>R) [18]} have been found in association with numerous diseases. All three mutations likely affect cofactor binding. An S695L mutation was identified in both WS [21] and ETP ALL [26]. This mutation may affect cofactor and substrate binding indirectly by influencing the conformation of residues in direct contact with these ligands.

A692 is located just after the terminus of α-5 (Figure 6) and has been found mutated in DLBCL (A>V)[23,33]. The backbone atoms of the residues in the analogous position in homologous structures are within van der Waals distance of both the cofactor and substrate. The backbone carbonyl oxygen of the structurally homologous residue (I1168) in euchromatic histone methyltransferase 1 (EHMT1) points directly towards the nexus of the substrate amine of the lysine and the methionyl group of the cofactor. Mutation of this residue therefore has the potential to affect the relative orientation of the substrate and cofactor in the EZH2-SET active state and affect the rate and substrate specificity of catalysis.

Mutation of N693 (Figure 6) has been identified in acute myelomonocytic leukemia (AMML) (N>T) [18]. The side chain of the structurally homologous residue in EHMT1 hydrogen bonds to the ribose of the cofactor. Thus, mutation of this residue is expected to affect cofactor binding in the active form of EZH2-SET. The EHMT1 residue following, H1170, interacts directly with the adenine moiety of the cofactor, and mutations of the structurally homologous residue in EZH2-SET, H694 (Figure 6), have been discovered in both WS (H>Y) [22] and CMML (H>R)[18].

#### Mutations outside the active site

**Figure 7:**
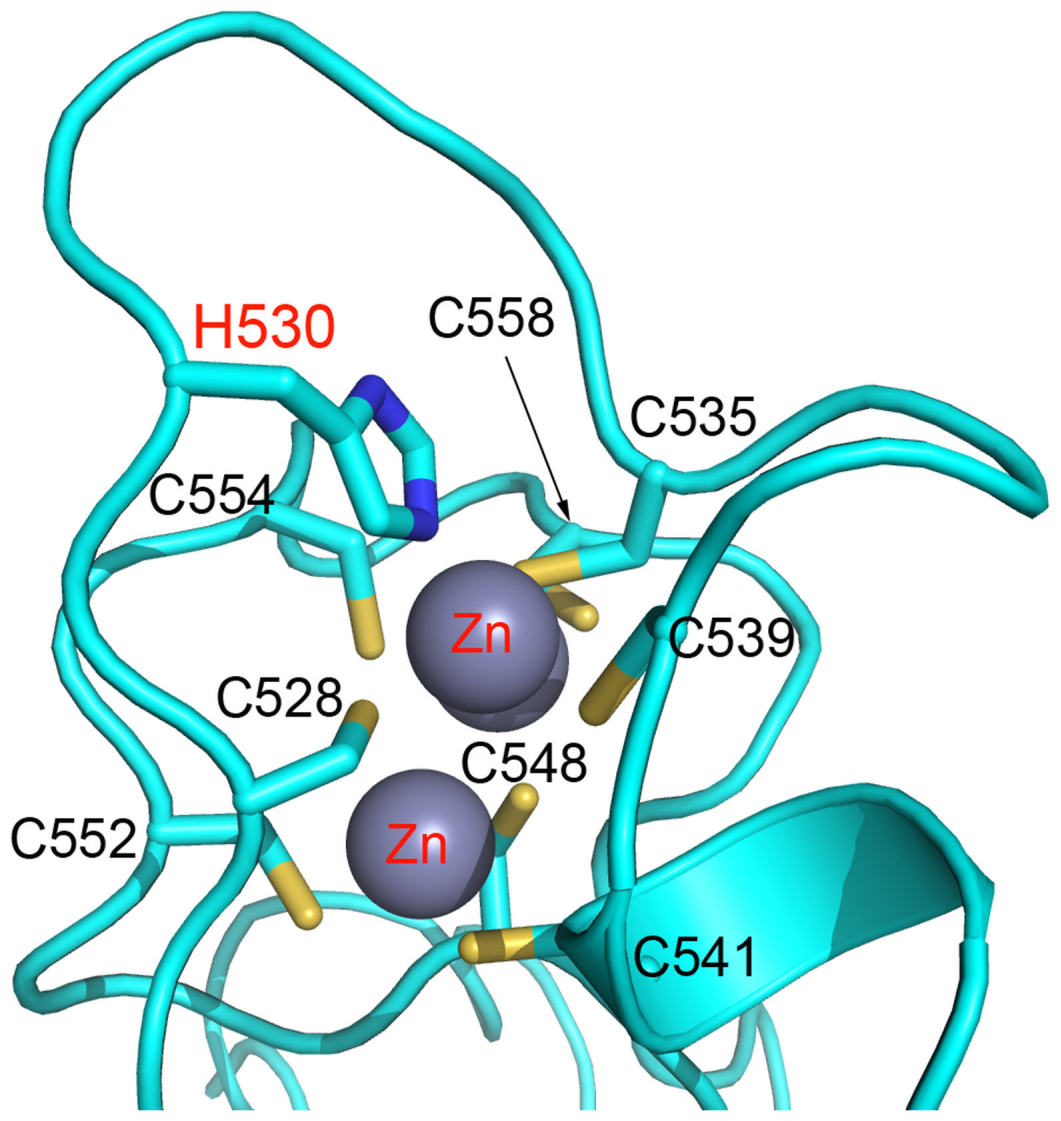
Location of mutation in the first zinc binding domain of EZH2-SET. EZH2-SET (cyan) is represented as a ribbon diagram with zinc atoms shown as gray spheres and side chain represented as sticks (carbon, cyan; nitrogen, blue; sulfur, sienna) A H530N mutation was identified in AML [29]. This mutation disrupts coordination of zinc in the first zinc binding domain likely having a strong destabilizing effect on the protein.

**Figure 8:**
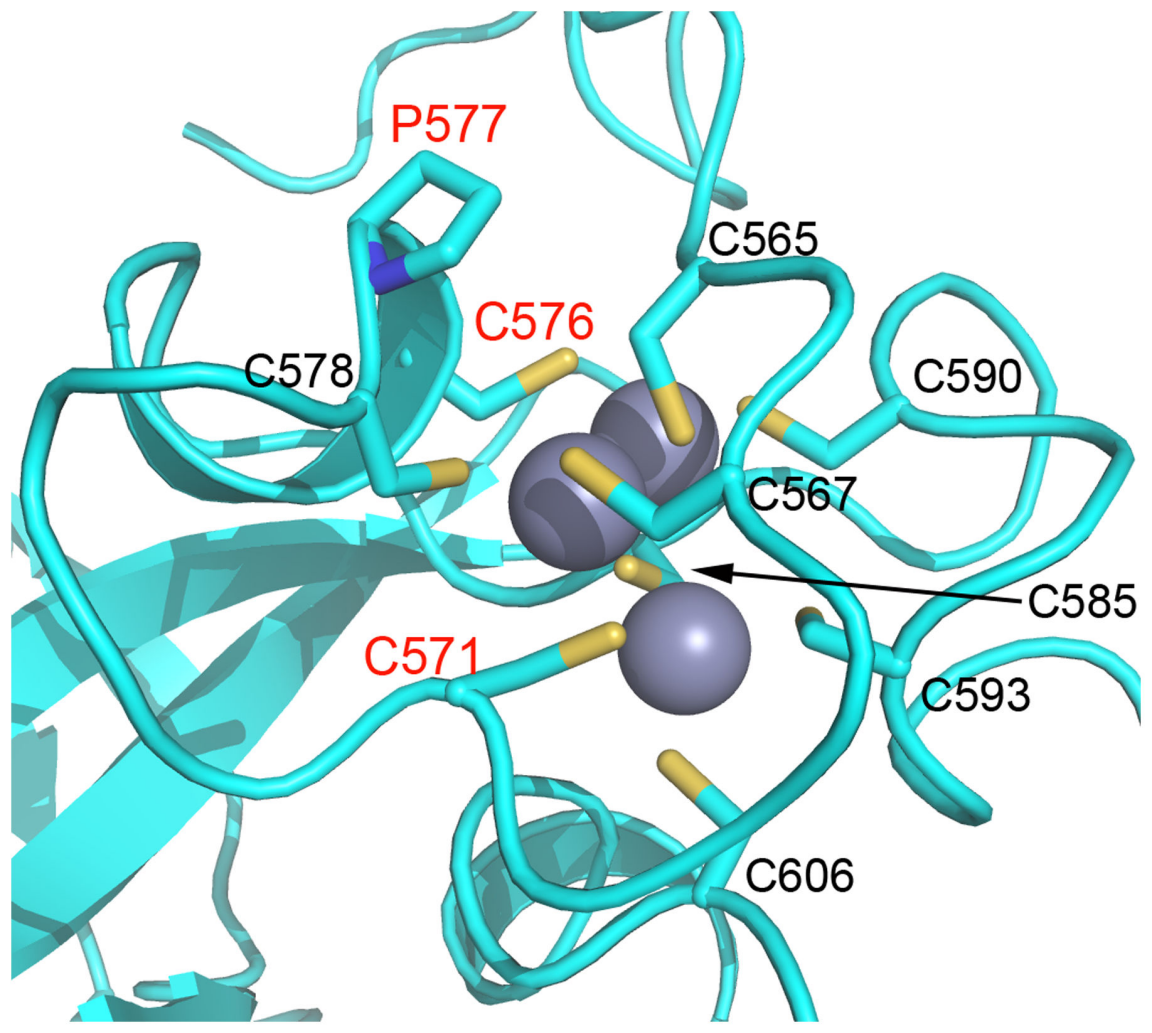
Location of mutation in the second zinc binding domain of EZH2-SET. EZH2-SET (cyan) is represented as a ribbon diagram with zinc atoms shown as gray spheres and side chain represented as sticks (carbon, cyan; nitrogen, blue; sulfur, sienna) A C571Y mutation was identified in MF [31] and a C576W mutation was identified in MDS [20]. These mutations disrupt coordination of zinc in the second zinc binding domain likely destabilizing the protein. Additionally, a P577L mutation was observed in ETP ALL [26].

Despite the inactive conformation observed in the EZH2-SET domain crystal structure, the biochemical consequences of numerous disease-associated mutations can be ascertained unambiguously. Several such mutations disrupt coordination of zinc atoms in the zinc binding domains. The H530N mutation which has been found in association with AML [29] disrupts coordination of zinc by histidine in the first zinc binding domain likely destabilizing the protein (Figure 7). The C571Y mutation found in myelofibrosis (MF) would similarly disrupt metal coordination in the second zinc binding domain [31](Figure 8). Another mutation (C576W) was identified in a case of MDS [20] and disrupts coordination of zinc by cysteine in the second zinc binding domain also likely having a strong destabilization effect (Figure 8). The P577L early T-cell precursor acute lymphoblastic leukemia (ETP ALL) mutation would also have a structurally destabilizing effect by altering the structure of the second zinc binding domain [26] (Figure 8).

**Figure 9:**
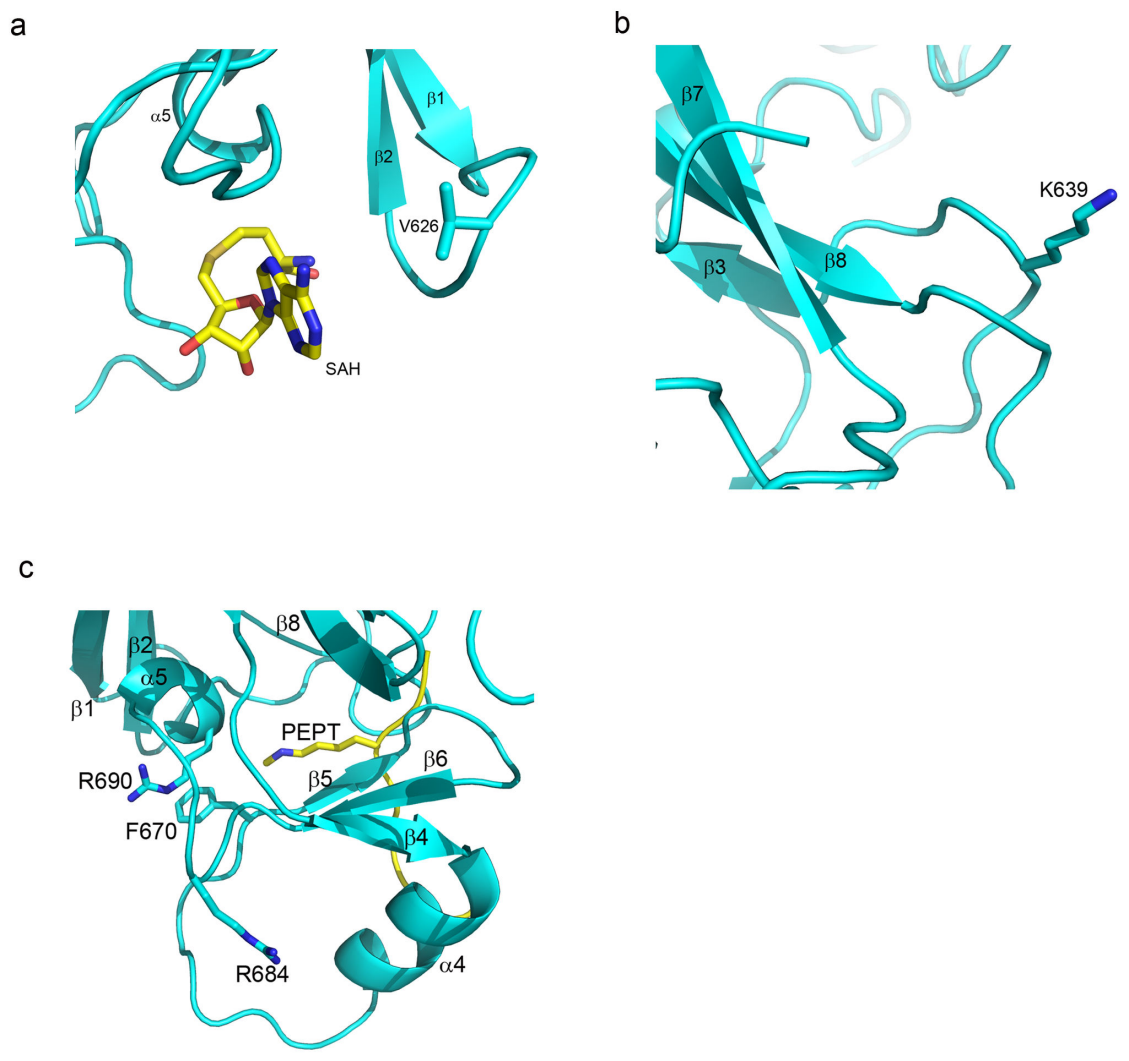
Additional disease-associated mutations outside this active site. The crystal structure of the EZH2-SET domain is represented as a ribbon model (cyan) with the hypothetical positions of cofactor and substrate (sticks colored by atom: carbon, yellow; oxygen, red; nitrogen, blue) extracted from the superimposed structure of EHMT1/PEPTIDE/SAH (PDB ID: 3HNA). EZH2-SET amino acid side chains are represented as sticks colored by atom (carbon, cyan; oxygen, red; nitrogen, blue). Secondary structure elements are labeled. (**a**) A V626M mutation was identified in WS [21]. This residue is located in the loop connecting β-1 and β-2 and may indirectly affect cofactor binding. (**b**) A K639E mutation was identified in WS [21]. This residue is located in the loop connecting β-2 and β-3. (**c**) R684 mutations were identified in WS (R>C) [21], ETP ALL (R>H) [26], and MF (R>C) [31]. This residue does not participate in cofactor or substrate binding; however, its side chain does pack against α-4 which does participate in substrate binding in homologous SET domains. R690 mutations were identified in CMML (R>H) [17,18] and MDS (R>C) [28]. This residue packs against F670 which in homologous SET domains contributes to the substrate lysine binding channel.

The V626M mutation identified in a WS [21] patient occurs in a solvent-exposed loop which connects β-strand β-1 and β-2 (Figure 9a). The structurally homologous loop in comparable SET domains helps to form the wall of the cofactor binding pocket that is opposite from the active site and, therefore, it can be surmised that this mutation may impair the active state of the EZH2-SET domain by compromising cofactor binding. The K639E WS mutation [21] is found in a solvent-exposed region preceding β-3 (Figure 9b). The side chain makes no critical contacts with the domain core so the phenotype associated with the mutation likely results from some subtle destabilizing effect on EZH2 or a disruptive effect upon its interaction with a critical binding partner.

Mutations of residue D664 have been observed in both MDS (D>E) [20] and WS (D>V) [21]. D664 is located in a solvent-exposed loop connecting α-5 and β-4 (Figure 2). The conformation of this residue is not well defined by electron density maps; however, the residue is clearly solvent exposed which could explain why its mutation to a hydrophobic residue such as valine would affect the protein’s stability and function. 

Mutations of A682, a buried residue found at the terminus of β-6, have been identified in cases of lymphoma (A>G)[27,33], WS with neuroblastoma (A>T)[21], and AML (A>V)[29]. Interestingly, A682 is packed against, and forms tight van der Waals interactions with the side chain of Y646 (3.6 Ǻ) (Figure 4), which itself was found mutated in cases of DLBCL [24]. It may be that mutation of A682 has an indirect effect on substrate specificity by influencing the conformation of the Y646 side chain, the ultimate functional affect being analogous to mutating the Y646 residue itself.

R684 has been found mutated in WS (R>C) [21], Acute Lymphoblastic Leukemia (ALL) (R>H) [26], and MF (R>C) [31]. R684 is located in the loop connecting β-6 and α-5 (Figure 9c), and its side chain extends into solvent with long range polar interactions (3.9-4.5 Ǻ) with numerous neighboring residues (E648, R659, 681). The guanidinium group of the R684 side chain is packed against the back face of the α-4 helix, the opposite face of which forms the left hand wall of the substrate binding groove. Mutation of this residue to one with a shorter side chain may affect the positioning of this helix. In addition, a solvent exposed cysteine at this position subject to oxidation may affect the protein’s stability.

Mutation of R690 has been identified in CMML (R>H) [17,18] as well as MDS (R>C) [28]. R690, the side chain of which is partially solvent-exposed, is located in the middle of α-5 at the base of EZH2-SET domain and does not directly impinge upon the active site (Figure 9c). However, R690 packs against the F670 side chain. The residue in this position contributes to substrate binding in homologous SET domains (EHMT1, Y1142), implying the possibility that mutation of R690 may indirectly affect substrate binding.

While based on homologous structures it would not be hypothesized that S695 interacts directly with the substrate or cofactor, this residue is located in the hypothetical cofactor binding loop connecting α-5 and β-7 (Figure 6), immediately following residues that, in analogous positions in the EHMT1, interact directly with the cofactor. It has been found mutated in WS (S>L) [21]. S695 is partially solvent exposed and therefore an S>L substitution would be unfavorable. Furthermore, mutations at this position would likely indirectly affect cofactor binding by influencing the conformations of neighboring residues. Interestingly, this same mutation was identified in a case of ETP ALL [26].

## Discussion

While the intricate hydrogen bonding network between the C-terminus and the domain core and the overall complementarity of the interaction suggest a possible mechanism of autoregulation, the possibility that the observed conformation is a crystallographic artifact cannot be disregarded. However, the fact that EZH2, absent its binding partners EED and SUZ12, does not engender biochemical activity implies that a conformational change occurs in the presence of accessory proteins, one which transforms EZH2 from an inactive to an active state. The crystal structure presented herein may represent the first of these two states. Considering what is known about the interactions between EZH2 and its obligate partners, the nature of how this interaction might affect the aforementioned transformation is speculative. EED is known to bind to an α-helix at the N-terminus of EHZ2 and, based on the interactions between homologous proteins, SUZ12 would be expected to bind to a region central to the EZH2 sequence. 

The conformational flexibility of the post-SET domain, and its role in regulating the activity of the SET-domain methyltransferases by controlling access to the substrate site, is well documented in multiple members of the family [55,56]. In the absence of the peptide substrate, the post-SET domain is often flexible and disordered in the crystal structures. If the present EZH2-SET domain crystal structure accurately reflects the biologically inactive state of EZH2, it would appear one of two, or a combination of two, processes must take place. Firstly, interactions with partners and ligands must induce a conformational change that allows the captured C-terminus to be released and subsequently recapitulate part, or all, of the deconstructed cofactor binding pocket. If this conformational event alone is not sufficient to generate a biochemically competent entity, then binding partners must directly contribute a presently undefined structural component that serves to reconstitute a catalytically active binding site. The absence of a cysteine rich zinc binding segment in the post-SET domain is also noted in the structures of SET7/9, SET8 and Rubisco methyltransferases. In those structures, the C-terminal post-SET domain forms part of the cofactor binding site without a zinc stabilized motif. It is likely that, in EZH2, a competent cofactor binding site is only formed upon binding to other components of the PRC2 complex. At least one specific protein-protein interaction involving the EZH2-SET domain has been characterized, that being with the XNP/ATR-X gene product [57]. 

In recent years, a plethora of studies have identified disease-associated genetic aberrations in the EZH2 coding sequence. The biological ramifications of these aberrations are varied and require further analysis and characterization. Both gain-of-function and loss-of-function mutations in EZH2 have been reported to result in clinical maladies. Perhaps the etiology of these diseases will eventually be understood in the context of EZH2 activity. However, deconvoluting the complex epigenetic code that regulates gene expression requires the simultaneous understanding of the countervailing forces that suppress and stimulate gene expression. The subtle balance between these opposing processes is underscored by seemingly contradictory findings that decreases in H3K27 trimethylation have been correlated with both improved and worsened prognosis in cancers [40,41,58]. The crystal structure of the EZH2-SET domain provides a framework for forming mechanistic hypotheses that explain the clinical phenotypes that arise from some of the known mutations of the EZH2 gene. Moreover, the crystal structure may provide critical insights into how EZH2 is activated by binding to its partners SUZ12 and EED. Still much regarding how EZH2 functions biologically is left unrevealed by the crystal structure of the isolated EZH2-SET domain and a more comprehensive understanding of EZH2 function would certainly be imparted by the crystal structure of the EZH2 in complex with EED and SUZ12. 

## Supporting Information

Figure S1 **Alignment of SET Domain amino acid Sequences.** The SET domains of EHMT1 (PDB:3HNA), SUV39H1 (PDB: 2R3A), and EZH2 are aligned. Critical residues of EHMT1 and SUV39H1 that contact the cofactor SAM are underlined. The C-terminal tail of the post-SET domain is boxed. Cysteines that bind zinc and stabilize the cofactor binding pocket are highlighted yellow. Identically conserved residues are orange. Conservative substitutions are blue. The residue numbering is derived from EZH2 isoform A.
(TIF)
