# Supplementary figures and images for: Structural Context of Disease-Associated Mutations and Putative Mechanism of Autoinhibition Revealed by X-Ray Crystallographic Analysis of the EZH2-SET Domain
Source: PLoS One. 2013 Dec 19;8(12):e84147. doi: 10.1371/journal.pone.0084147 (PMC3868555; doi:10.1371/journal.pone.0084147)

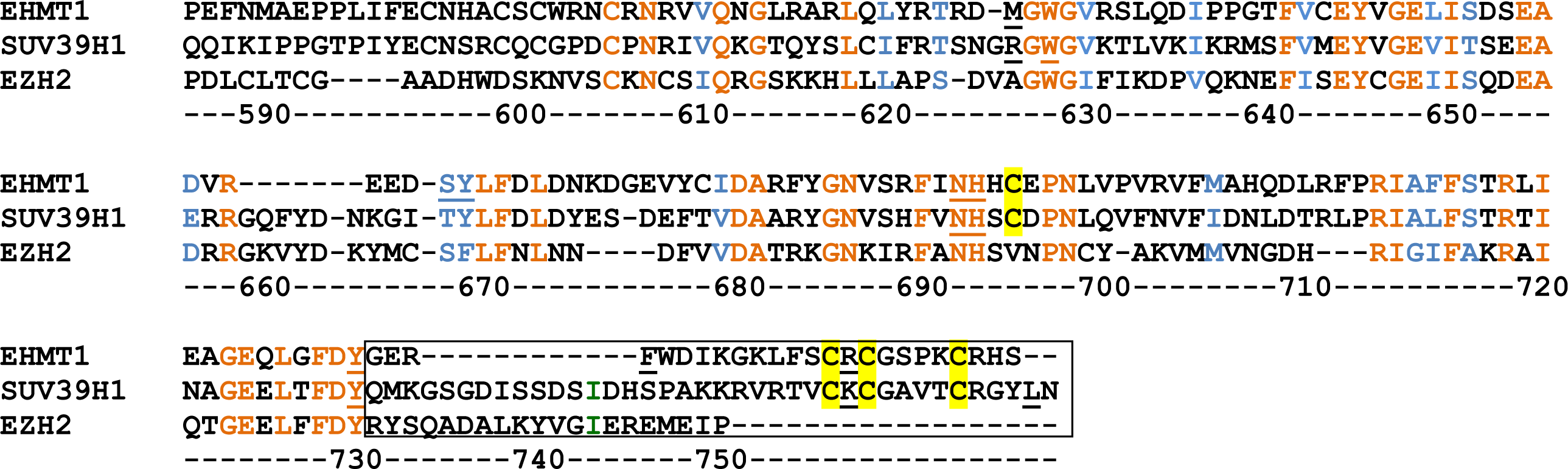

Supplement: Figure S1 — Alignment of SET Domain amino acid Sequences. The SET domains of EHMT1 (PDB:3HNA), SUV39H1 (PDB: 2R3A), and EZH2 are aligned. Critical residues of EHMT1 and SUV39H1 that contact the cofactor SAM are underlined. The C-terminal tail of the post-SET domain is boxed. Cysteines that bind zinc and stabilize the cofactor binding pocket are highlighted yellow. Identically conserved residues are orange. Conservative substitutions are blue. The residue numbering is derived from EZH2 isoform A. (TIF) [file pone.0084147.s001.tif]
